# Intersections of Gender and Race among Older Workers during the COVID-19 Pandemic

**DOI:** 10.1093/geroni/igab046.3150

**Published:** 2021-12-17

**Authors:** Takashi Yamashita, Wonmai Punksungka, Samuel Van Vleet, Abigail Helsinger, Phyllis Cummins

**Affiliations:** 1 University of Maryland, Baltimore County, Baltimore, Maryland, United States; 2 Miami University, Oxford, Ohio, United States; 3 Scripps Gerontology Center, Miami University, Oxford, Ohio, United States

## Abstract

The novel coronavirus/COVID-19 pandemic in 2020 has impacted the aging workforce. In addition to local data and case studies that are rapidly increasing, baseline national-level inquiries are needed for investigating relevant social inequalities. Also, the intersections of gender and race/ethnicity among older adults are critical yet understudied areas. For example, older minority women’s experience in the pandemic, compared to older men, are yet to be investigated. We analyzed the nationally representative 2020 Health and Retirement Study (HRS) COVID-19 module data. Based on the sample of 2,086 adults aged 50 years and older, employment during the pandemic as well as psychosocial measures, including social support and attitudes toward work, are examined. We used survey-weighted binary logistic regression models. Results showed that older Black women (Odds-ratio = 0.52, p = 0.02 < 0.05) were less likely to work for pay compared to White women during the pandemic. Also, older Hispanic men (Odds-ratio = 2.82, p = 0.03 < 0.05) were more likely to work for pay than older White men. Older Hispanic women (Odds-ratio = 2.41, p = 0.03 < 0.05) were more likely to worry about getting social support during the pandemic than White women. However, there was no significant differences in the changes in attitudes toward work across gender and racial/ethnic groups during the pandemic. Based on the baseline national data analysis, we discussed possible policy changes and interventions that consider the intersections of gender and race/ethnicity to help older adults re-adjust to post-pandemic work environments and labor markets.

